# Impact of COVID-19 Pandemic on Remote Monitoring of Cardiac Implantable Electronic Devices in Italy: Results of a Survey Promoted by AIAC (Italian Association of Arrhythmology and Cardiac Pacing)

**DOI:** 10.3390/jcm10184086

**Published:** 2021-09-10

**Authors:** Massimiliano Maines, Pietro Palmisano, Maurizio Del Greco, Donato Melissano, Silvana De Bonis, Stella Baccillieri, Gabriele Zanotto, Antonio D’Onofrio, Renato Pietro Ricci, Roberto De Ponti, Giuseppe Boriani

**Affiliations:** 1 Santa Maria del Carmine Hospital, 38068 Rovereto, Italy; massimiliano.maines@apss.tn.it (M.M.); Maurizio.DelGreco@apss.tn.it (M.D.G.); 2 Cardiology Unit, “Card. G. Panico” Hospital, 73039 Tricase, Italy; 3 F. Ferrari Hospital, 73042 Casarano, Italy; donato@melissano.org; 4 Department of Cardiology, Ospedale “Ferrari”, 87012 Castrovillari, Italy; silvanadebonis68@gmail.com; 5 Ospedale San Bassiano, 36061 Bassano del Grappa, Italy; hhbacci@gmail.com; 6 Ospedale Mater Salutis Legnago, 37045 Verona, Italy; gabriele.zanotto62@gmail.com; 7 Electrophysiology and Cardiac Pacing Unit, A.O.R.N, Ospedali dei Colli-Monaldi, 80131 Naples, Italy; donofrioant1@gmail.com; 8 Centro CardioAritmologico, 00152 Roma, Italy; renatopietroricci@gmail.com; 9 Department of Medicine and Surgery, University of Insubria, 21100 Varese, Italy; roberto.deponti@uninsubria.it; 10 Department of Biomedical, Metabolic and Neural Sciences, Cardiology Division, University of Modena and Reggio Emilia, Policlinico di Modena, 41121 Modena, Italy; giuseppe.boriani@unimore.it

**Keywords:** COVID-19, remote monitoring, telecardiology, cardiac implantable electronic devices

## Abstract

The COVID-19 pandemic has had a profound impact on the organisation of health care in Italy, with an acceleration in the development of telemedicine. To assess the impact of the COVID-19 pandemic on the spread of remote monitoring (RM) of cardiac implantable electronic devices (CIEDs) in Italy, a survey addressed to cardiologists operating in all Italian CIED-implanting centres was launched. A total of 127 cardiologists from 116 Italian arrhythmia centres took part in the survey, 41.0% of all 283 CIED-implanting centres operating in Italy in 2019. All participating centres declared to use RM of CIEDs. COVID-19 pandemic resulted in an increase in the use of RM in 83 (71.6%) participating centres. In a temporal perspective, an increase in the median number of patients per centre followed up by RM was found from 2012 to 2017, followed by an exponential increase from 2017 to 2020. In 36 participating centres (31.0%) a telehealth visits service was activated as a replacement for in-person outpatient visits (in patients with or without CIED) during the COVID-19 pandemic. COVID-19 pandemic has caused an acceleration in the use of RM of CIEDs and in the use of telemedicine in the clinical practice of cardiology.

## 1. Introduction

The COVID-19 pandemic has had a profound impact on the organisation of health care in Italy, with a drastic reduction in hospital access and traditional controls [1]. Many cardiology departments were actually closed to be converted into COVID-19 departments. As a consequence, a wider application of telecardiology was promoted, in order to follow cardiac patients at distance [2]. Remote monitoring (RM) of cardiac implantable electronic devices (CIEDs) by means of telemonitoring allows technical information to be obtained on the operation of the device—avoiding patients having to access the hospital for checking—and clinical information on the patient’s health conditions, for example on the degree of compensation with a good ability to prevent events of this type.

In consideration of the need to assess the impact of the COVID-19 pandemic on the spread of RM of CIEDs and telecardiology in Italy, the Italian Association of Arrhythmology and Cardiac Pacing (AIAC) promoted a survey among Italian arrhythmia centres.

## 2. Methods

From 13 July 2020 to 25 February 2021 a survey endorsed by the AIAC was published on the official AIAC website (https://aiac.it/, accessed on 13 November 2020). The survey was open to cardiologists operating in all Italian cardiac CIED-implanting centres. Participation in the survey was voluntary. The questionnaire could be completed by more than one cardiologist from the same centre. The questionnaire consisted of 33 questions: seven of them focused on the characteristics of the participating centres (i.e., device implantation volume and number of in-office CIED follow-up examinations per year); nine of them focused on the use of RM (i.e., number of patients enrolled in the RM follow-up programme, type of RM systems used, type of CIED in which RM is used, characteristics of patients followed up by RM, main purpose of RM, impact of RM on the frequency of in-office device follow-up visits); eight of them focused on the used organizational model (i.e., subjects involved, rate of transmission reviews, professionals assigned to primary review of remote data, in which cases transmissions are reviewed by the physician); seven of them focused on the impact of COVID-19 pandemic on the use of RM (i.e., change in number of patients followed up by RM, activation of a telehealth visit service); and the remaining two were focused on future perspectives. Twenty-eight of the 33 questions were multiple-choice questions (see online Supplementary Materials for details).

All respondents agreed to participate in the research. The data are reported in grouped format (counts and percentages), such that participants are not able to be identified from the results. In addition, the survey involved the use of records that contained only non-identifiable data about the participants. For these reasons, the study has not been reviewed or approved by a human research ethics committee.

### Statistical Analysis

Descriptive statistics were reported as means and standard deviations for normally distributed continuous variables. Continuous variables with skewed distribution were reported as medians and 25–75th percentiles. The Student’s t-test or the Mann–Whitney U test was used to compare continuous variables between groups. Categorical variables were reported as percentages and compared using the χ^2^ test or Fisher’s exact test, as appropriate. *p* values < 0.05 were considered statistically significant. 

## 3. Results

### 3.1. Participating Centres

**Figure 1:**
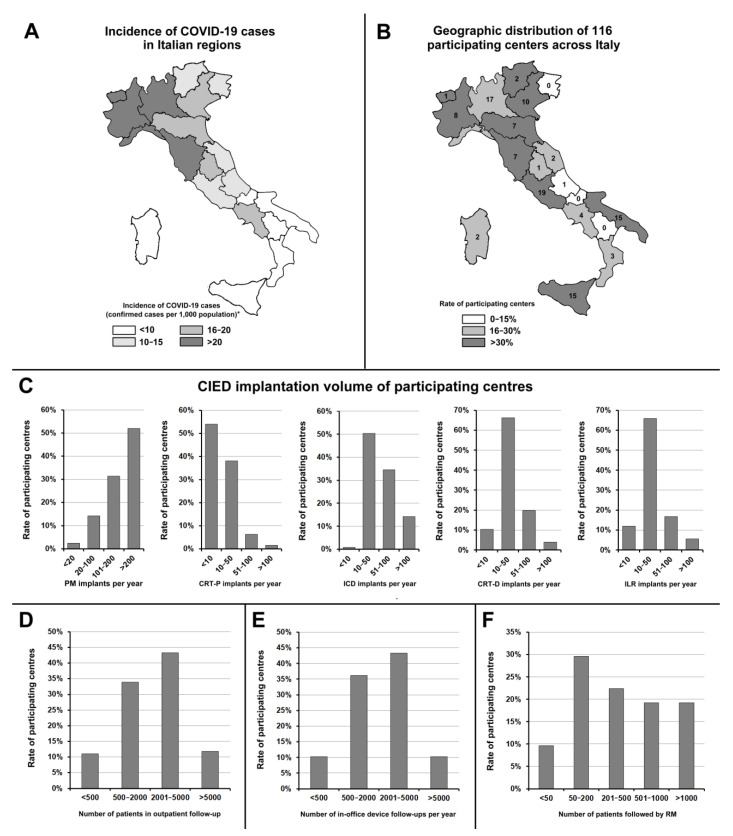
(**A**) Incidence of COVID-19 cases in Italian regions. (**B**) Geographic distribution of the centres that responded to the survey across Italy. (**C**) CIED implantation volume of participating centres. (**D**) Number of patients in outpatient follow-up. (**E**) Annual number of in-office device follow-up examinations performed by the participating centres. **(F**) Number of patients followed up by remote monitoring. * Data from Italian Civil Protection Department [4]. CIED, cardiac implantable electronic device; CRT-D, cardiac resynchronisation therapy combined with defibrillator; CRT-P, cardiac resynchronisation therapy combined with pacemaker; ICD, implantable cardioverter-defibrillator; ILR, implantable loop recorder; PM, pacemaker; RM, remote monitoring.

A total of 127 cardiologists from 116 Italian arrhythmia centres took part in the survey. For eight centres, more than one cardiologist responded to the survey (median: 2; range: 2–4). A complete list of participating centres is reported in Appendix. The centres which participated in the survey accounted for 41.0% of all 283 CIED-implanting centres operating in Italy in 2019 [3]. The participating centres displayed a wide geographical distribution (Figure 1B): a median of three centres per region (range: 0–19; interquartile range: 1–10) responded. In eight regions, there were five or more participating centres. The response rate was similar in Northern, Central and Southern Italy (38.8, 51.9, and 38.1% of all operating centres, respectively, *p* = 0.206). After dividing the Italian regions into four groups, according to incidence of COVID-19 cases (confirmed cases <10, from 10 to 15, from 16 to 20, and > 20 per 1000 population, Figure 1A) [4], the response rate was similar in the regions with higher incidence of COVID-19 cases (confirmed cases > 20 per 1000 population, *n* = 5) compared to other regions (*n* = 15) (36.4 vs. 43.6%; *p* = 0.277).

Figure 1C shows the implantation volumes of pacemakers (PMs), implantable cardioverter-defibrillators (ICDs), cardiac resynchronisation therapy (CRT) devices, and implantable loop recorders (ILRs) of the participating centres. Many centres (43.3%) had from 2001 to 5000 CIED patients in outpatient follow-up (Figure 1D), and many centres (43.3%) declared to perform from 2001 to 5000 in-office device follow-ups per year (Figure 1E).

### 3.2. Use of Remote Monitoring

All participating centres declared that they used RM. Figure 1F shows the number of patients followed up by RM in participating centres. Many centres (29.6%) followed up by RM from 50 to 200 patients; 10.0% followed up by RM < 50 patients and 19.2% > 1000 patients.

Regarding the RM systems provided by the six manufacturers available in Italy at time of the survey, the most widely used was the CareLink Network (Medtronic Inc., Minneapolis, MN, USA) (91.3% of participating centres), followed by Latitude Patient Management System (Boston Scientific, St Paul, MN, USA) (87.4%), Home Monitoring system (Biotronik Gmbh, Berlin, Germany) (80.3%), Merlin.net (Abbott Inc., St Paul, MN, USA) (74.8%), Smartiview (MicroPort, Clamart, France) (29.1%), and Ermes (Medico S.p.A., Rubano, Italy) (7.1%).

**Figure 2:**
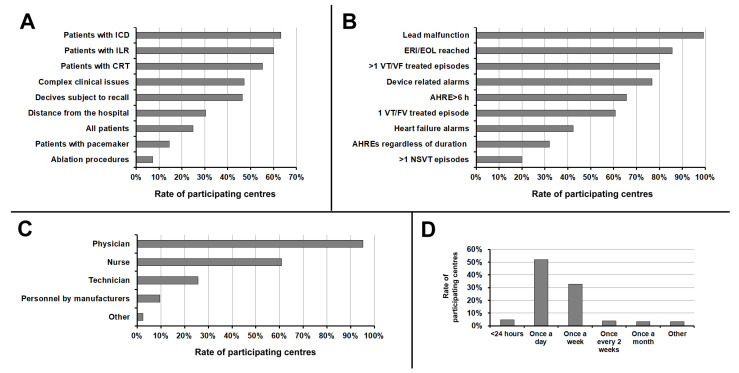
(**A**) Rate of use of remote monitoring according to type of CIED and patients’ characteristics. (**B**) Events considered as critical alerts to remote monitoring by the participating centres. (**C**) Professionals involved in the management of remote monitoring. (**D**) Frequency with which remote transmissions were reviewed. AHRE, atrial high rate episode; CIED, cardiac implantable electronic device; CRT, cardiac resynchronisation therapy; EOL, end-of-life; ERI, elective replacement indicator; ICD, implantable cardioverter-defibrillator; ILR, implantable loop recorder; NSVT, nonsustained ventricular tachycardia; PM, pacemaker; RM, remote monitoring; VF, ventricular fibrillation; VT, ventricular tachycardia.

Regarding the type of CIED, RM was more frequently implemented in patients with ICDs, followed by ILRs, CRT devices, and PMs. Concerning patient characteristics, RM was more frequently used in patients with complex clinical issues, followed by those with CIEDs at risk of malfunction (subject to recall), those who lived far from the hospital, and those undergoing arrhythmia ablation procedures (Figure 2A).

The main purpose of RM reported by the vast majority of participating centres (87.9%) was periodic, scheduled remote device interrogation, in addition to automatic alerts for device/lead malfunction and for clinical events (such as ventricular or atrial tachyarrhythmias). The remaining centres reported periodic remote device interrogation in addition to automatic alerts for device/lead malfunction (8.1%) or the complete replacement of in-office device checks (4.0%) as the main purpose of RM.

Figure 2B shows which events were considered as critical alerts to RM by the participating centres. The majority of centres (>75%) considered critical alerts to be lead malfunction, battery depletion reached (elective replacement indicator/end-of-life), >1 ventricular tachyarrhythmia treated episode, and device-related alarms (i.e., non-physiological variations in impedance/pacing threshold/sensing). More rarely (<50%), heart failure alarms, atrial high rate episodes detection, and the detection of >1 non-sustained ventricular tachycardia episode were considered as critical alerts.

Regarding the impact of RM on the frequency of in-office device follow-up visits, a significant reduction in the number of in-office examinations of PM, ICD and CRT patients was reported by 60.8, 68.0 and 65.6% of responding centres, respectively. Specifically, in PM, ICD and CRT patients followed up by RM, in-office device follow-up examinations were planned once a year in 39.2, 58.4 and 55.2% of centres, respectively, and less than once a year in 21.9, 9.6 and 10.4%, of respondents, respectively.

### 3.3. Organisation of Remote Monitoring

The vast majority of responding centres (95.2%) reported that one or more physicians were involved in the management of RM. The second most frequently involved professional was the nurse (60.8%), followed by technician (25.6%), and technical personnel employed by manufacturers (9.6%) (Figure 2C). The personnel provided by manufacturers was always employed in addition to hospital personnel, as a technical support. The professionals most frequently assigned to primary remote data review were nurses (43.3% of participating centres) and physicians (36.2%), followed by technicians (20.5%). When the primary review of transmissions was not performed by the physician, the transmissions were submitted to the physician only in case of critical events in 67.8% of the centres; in the remaining 32.2% of the centres, transmissions were submitted to the physician in all cases. 

Figure 2D shows the frequency with which transmissions were reviewed. The majority of the centres (52.0%) declared that transmissions were reviewed once a day (only on working days), and in 32.8% of the centres, once a week.

Most centres (63.7%) reported that the results of remote transmissions were usually communicated to patients only in the case of clinically significant events. The results were communicated to patients by telephone in the majority of the centres (60.0%), by email or letter in the remaining centres.

The majority of participating centres (78, 67.2%) declared that they did not usually share the clinical data collected by RM with other medical specialists. Twenty-one of the remaining 38 centres (55.3%) declared that they communicated the remote clinical data to the attending cardiologist; 15 (39.5%) declared that they shared the remote clinical data with other specialist clinics; nine (23.7%) declared that they communicated the results of transmissions indicating clinically significant events to the family doctor. The results of remote transmissions were transmitted to other medical specialists when a critical clinical event is detected in 54.8% of centres, with a scheduled frequency (every 3, 6 or 12 months) in the remaining centres. Only 21.1% of the participating centres had an electronic health record to record remote clinical data and to make them available to all professionals involved in patient management.

### 3.4. Impact of COVID-19 Pandemic on the Use of Remote Monitoring

**Figure 3:**
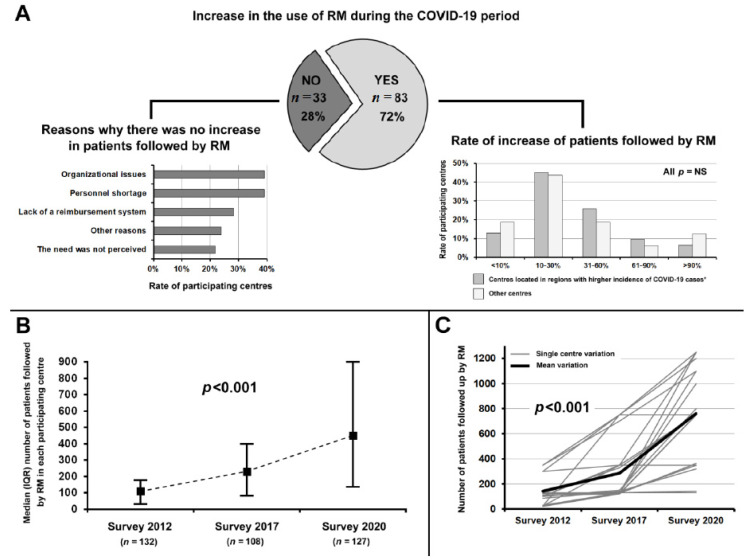
(**A**) Rate of participating centres reporting or not an increase in the use of remote monitoring during the COVID-19 pandemic; reasons why there was no increase in the number of patients followed up by remote monitoring (graph on the right); rate of increase in patients followed up by remote monitoring (graph on the left). (**B**) Median number of patients per centre followed up by remote monitoring in 2012, 2017 and 2020 surveys. (**C**) Variation in the number of patients followed up by remote monitoring from 2012, 2017 and 2020 in the centres which participated in all three surveys. * Data from Italian Civil Protection Department [2]. IQR, interquartile range; NS, not significant; RM, remote monitoring.

The COVID-19 pandemic has resulted in an increase in the use of RM in 83 (71.6%) participating centres (Figure 3A). The rate of the participating centres located in regions with higher incidence of COVID-19 cases (confirmed cases > 20 per 1000 population) that reported an increase in the use of RM was similar to that of the participating centres located in other regions (79.4 vs. 68.3%; *p* = 0.227). In many centres (44.2%), the number of CIED patients followed up by RM increased from 10 to 30%, in 39.0% of centres it increased >30%, and in the remaining 17.8% it increased <10%. No significant difference was found in the amount of increase in the centres located in regions with higher incidence of COVID-19 cases compared to the other centres (Figure 3A). Thirty-three participating centres (28.4%) reported no increase in the use of RM as a result of the COVID-19 pandemic. The most frequent reasons for the lack of an increase in the number of patients followed up by RM reported by these centres were organizational issues and personnel shortage, followed by the lack of a tariff for reimbursement of RM and the lack of perception of the need (Figure 3A).

In 2012 and 2017, the AIAC promoted two surveys on RM in which 132 and 108 Italian centres participated, respectively [5]. Twenty-one centres took part in the 2012, 2017 and 2020 surveys. As shown in Figure 3B, there was an increase in the median number of patients per centre followed up by RM from 2012 to 2017, and an exponential increase from 2017 to 2020. Considering only the 21 centres that participated to all three surveys, the upward trend was confirmed (Figure 3C).

In 36 participating centres (31.0%) a telehealth visits service was activated as a replacement for in-person outpatient visits (in patients with or without CIED) during the COVID-19 pandemic. In most of these centres (75.0%) this service will also be maintained in 2021. Telehealth visits were performed most frequently by phone calls (70%), followed by video calls (25.0%), and by dedicated software (22.7%).

In 17 participating centres (14.7%) a teleconsulting system was activated for the family doctor or for physicians of other centres during the COVID-19 pandemic.

**Figure 4:**
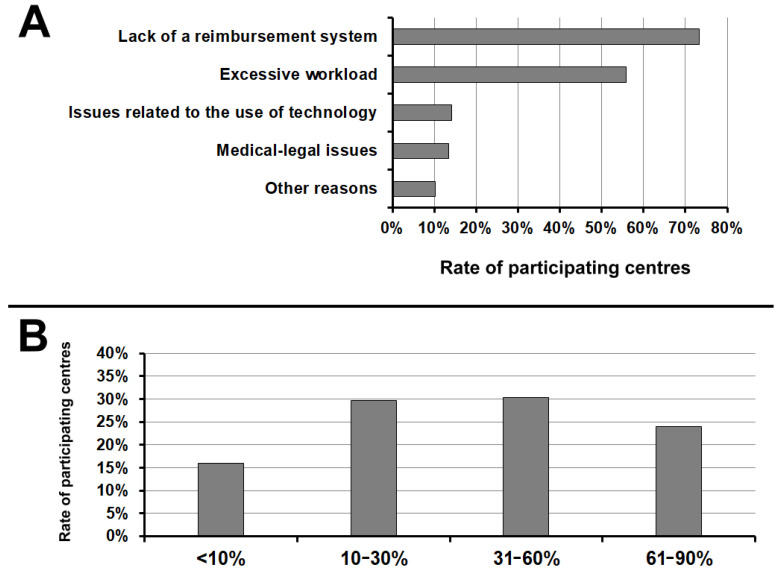
(**A**) Main barrier to the implementation of remote monitoring. (**B**) Rate CIED patients who will be followed up by remote monitoring in the next 5 years. CIED, cardiac implantable electronic device.

For the majority of participating centres (73.2%) the main barrier to the implementation of RM was the lack of a tariff for reimbursement, followed by the excessive workload (55.9%), issues related to the use of technology (14.2%), medical/legal issues (13.4%), and other reasons (10.2%) (Figure 4A).

Finally, the vast majority of respondents (84.0%) hypothesised that in the next 5 years >30% of CIED patients will be followed up by RM (Figure 4B).

## 4. Discussion

This survey collected data from about a third of the arrhythmia centres operating in Italy, with a geographical distribution of responses that covers the entire national territory and is independent of the local impact of COVID-19 infection. It provides a representative picture of the use and management of RM of CIEDs in current Italian clinical practice and insights on the impact of the COVID-19 pandemic.

This survey confirms that in Italy most of the implantation centres manage mid volumes of patients [5]. All participating centres declared to have adopted RM, but the median of patients followed is 50–200 with less than 20% of centres using RM extensively (in more than 1000 patients), regardless of what was recommended in the 2015 European Heart Rhythm Association (EHRA) consensus document [6].

RM continues to be offered mainly to patients with ICDs with or without CRT, in particular to those with devices at risk of malfunction, with complex clinical problems or residing far from the hospital. The other category of patients in which RM is used extensively are patients with ILRs, in which this type of follow-up allows them to reduce office visits and to shorten diagnosis and therapy times [7]. In agreement with previous studies, implementation of RM significantly reduced the rate of in-office visits and the total use of healthcare resources [8].

The majority of centres (>75%) considered lead malfunction alert as critically important. The alert for battery depletion (elective replacement indicator/end-of-life) was identified as the second most important alert. Indeed, RM is effective in extending CIEDs longevity by reducing inappropriate shocks [9], allowing optimal device setting [10], and identifying the elective replacement indicator without increasing the rate of in-office visits [11].

As regards the organizational model, the nurse and less frequently the cardiology technician play a primary role and support physicians in managing the process, as suggested in the AIAC 2020 document [12]. The less frequent involvement of technicians is probably linked to the limited number of these professionals in the staff of Italian hospitals. The employees of manufacturers provide technical support to the hospital personnel involved in remote control in 9.6% of the centres, a proportion that will decline as soon as hospital nurses and technicians become fully confident with the technical aspects of CIEDs telemonitoring, as planned in the AIAC document [12].

The management model in which the nurse initially evaluates the transmissions is the most used in the Italian practice. In the majority of the participating centres, the transmissions are submitted to the referring physician in charge of the service only in case of critical events. In a previous work, Maines et al. [13] described the implementation of the organizational model proposed by AIAC, and measured the healthcare resources needed for its management; in that study, 39% of transmissions had events and only 20% required medical supervision. It would also be important to reduce unnecessary transmissions and optimise device programming so as to avoid redundant transmissions or alarms [14].

The availability of data from scheduled remote device interrogations and automatic notifications are known to allow prompt detection of clinical events [15], reducing the time to a clinical decision [16]. Expert guidelines suggest that patients should be educated as to the limitations of the technology (delay in transmission, schedule of data review, etc.), what they can reasonably expect from it, and the fact that it is not an emergency alert system [6]. Nonetheless, guidelines also point out that critical alerts should be communicated to the patient and acted on in a time-frame commensurate with the clinical significance of the finding. In current practice, the response times to the transmissions seem satisfactory, with more than 80% of the centres responding within a week, and 52% of the centres performing daily evaluations of the transmissions. This is important to manage technical alert condition, but also clinical notifications. For example, RM of CIEDs capable of continuous detection and characterisation of atrial arrhythmias over long periods allows to stratify the patients’ risk of ischemic stroke. Indeed, it has been shown that a maximum daily atrial fibrillation burden of 6 h is associated with an increase in the relative risk of stroke by 17% [17]. Thus, the use of atrial arrhythmias notifications may constitute an effective tool for taking into consideration antithrombotic treatment, even despite the lack of strong evidence and the many areas of uncertainty [18,19,20].

Most centres (63.7%) reported that the results of remote transmissions were usually communicated to patients only in the case of clinically significant events, and in most cases the results were communicated to patients by telephone. The results were communicated by email or letter more rarely. This can be a problem in many centres because communication via telephone or e-mail does not respect adequate standards of quality and privacy. It would be desirable to have a greater degree of computerisation in the future with the possibility that the reports are inserted in a patient file, visible to all the figures involved in the patient management. This already happens in some regions with positive results [13].

Despite the documented clinical and organizational benefits of RM, the policies for reimbursement of benefits by health systems and insurances vary significantly in different countries [21] and this represents a limit to their use on a large scale. In Europe, the situation is extremely heterogeneous and constantly evolving. According to a recent survey by the EHRA [22], the main barrier to the adoption of RM is the lack of reimbursement in many countries (in up to 88% of centres implanting devices). For the majority of participating centres (73.2%), the main barrier to the implementation of RM in Italy remains the lack of a reimbursement system, followed by the excessive workload (55.9%). This confirms the results of previous surveys. Nonetheless, according to the present results, it seems that the situation at a country level is improving, since a tariff for reimbursement system was reported as available by 41% of the centres interviewed.

During the recent COVID-19 outbreak, Italian health authorities mandated to replace in-person outpatient activities with remote evaluations [23,24]. Although this strategy aimed to minimise the risk of infection [25], the implications for the health of patients with cardiovascular diseases may potentially be unfavourable [26,27,28].

In a recent nationwide survey endorsed by the AIAC [1], most of the participating centres reported a reduction of >50% in the number of implants of CIEDs and of >50% in the number of ablation procedures performed in both elective and emergency setting during the COVID-19 outbreak. In agreement with this survey, a reduction of >50% in urgent pacemaker implants for severe bradyarrhythmias was reported by analysis performed on a single hospital basis or on a regional basis [29,30]. The COVID-19 outbreak also had a significant impact on the incidence of cardiovascular events. In a recent study, a 52% increase in the occurrence of out of hospital cardiac arrests was reported in some provinces from Lombardy in the first 2 months of the pandemic, and this increase was associated with worse in hospital outcomes [31]. On the other hand, in a recent study, a 32% reduction in ventricular arrhythmia burden was observed in a population of ICD patients remotely monitored [32]; the reduction in ventricular arrhythmias coincided with measures of social isolation, suggesting a potential role for real-life stressors in ventricular arrhythmia burden in ICD patients.

In our survey, an increase in the use of RM was reported in 72% of participating centres, and this was independent of the impact of COVID-19 in the hospitals that responded.

The implementation of RM of CIEDs in the last 3 years documented in this survey was greater than in the five-year period 2012–2017, which was found both when evaluating the responses of all the centres and when evaluating the progress in the centres that responded to all three surveys.

These data are partly linked to a natural spread of this clinical practice, but an acceleration seems to have come following the COVID-19 pandemic.

Several studies have shown that RM provides significant benefits to CIED patients, including the early detection of technical issues [33,34], and of atrial and ventricular arrhythmias [9,35,36,37]. In addition, several studies have evaluated the usefulness and effectiveness of device-based sensors in the early detection of worsening heart failure [38,39,40]. In addition, RM appears to have a positive effect on hard clinical endpoints, such as mortality and hospitalisation [41,42]. The exponential increase in the use of RM of CIEDs observed in the last 3 years in Italy, probably partly related to the COVID-19 pandemic, suggests that an increasing number of patients will benefit from this technology, and it is possible that this will have a positive impact on the cardiovascular outcomes of many CIED patients.

We hope that the use of MR will further increase over time given the numerous studies that support the advantages of RM of CIEDs and the recommendations that since 2015 put RM of devices in class I.

### Study Limitations

Only 116 out of 283 CIED-implanting centres operating in Italy took part in the survey (41.0% of the Italian centres). For this reason, our findings should be interpreted with caution as they could not accurately reflect the Italian clinical practice. However, the participating centres represent the clinical practice of high volume centres in terms of CIED implant with more than 100 CIED implanted per year in more than 80% of the participating centres.

Twenty-eight of the 33 questions of the questionnaire were multiple-choice questions. Multiple-choice questionnaires have some limitations. Respondents are required to choose a pre-made answer that does not exactly reflect their answer. Moreover, the subjective design of a multiple choice questionnaire may not allow to obtain a complete and detailed representation of the clinical reality

## 5. Conclusions

The COVID-19 pandemic has caused an acceleration in the implementation in Italy of RM of CIEDs. The figure at the centre of the organizational model is a dedicated nurse/technician with the support of the doctor, as also proposed by the AIAC consensus document. With the pandemic, telemedicine models for the remote management of patients without devices also had some promotion for its implementation.

## Data Availability

The data presented in this study are available upon reasonable request from the corresponding author. The data are not publicly available due to privacy restrictions.

## Supplementary Materials

The following are available online at https://www.mdpi.com/article/10.3390/jcm10184086/s1, Questionnaire sent to the centres.
