# Supplementary material for: Impact of COVID-19 Pandemic on Remote Monitoring of Cardiac Implantable Electronic Devices in Italy: Results of a Survey Promoted by AIAC (Italian Association of Arrhythmology and Cardiac Pacing)
Source: J Clin Med. 2021 Sep 10;10(18):4086. doi: 10.3390/jcm10184086 (PMC8469719; doi:10.3390/jcm10184086)
Supplement: Supplementary file 1 [file jcm-10-04086-s001.zip › jcm-1329134-supplementary.pdf]

Supplemenatry Materials
Questionnaire sent to the centres

Characteristics of the centre

1) Annual number of PM implants:
A) <20
B) 20-100
D) 101-200
E) >200

2) Annual number of CRT-P implants:
A) <10
B) 10-50
D) 51-100
E) >100

3) Annual number of ICD implants:
A) <10
B) 10-50
D) 51-100
E) >100

4) Annual number of CRT-D implants:
A) <10
B) 10-50
D) 51-100
E) >100

5) Annual number of ILR implants:
A) <10
B) 10-50
D) 51-100
E) >100

6) Number of CIED patients in outpatient follow-up:
A) <500
B) 500-2000
D) 2001-5000
E) >5000

7) Number of in-office device follow-up examinations performed per year (PM, CRT-P, ICD, CRT-D and ILR):
A) <500
B) 500-2000
D) 2001-5000
E) >5000

<u>Use of remote monitoring</u>

8) Is remote monitoring used in your centre?
   A) Yes
   B) No

9) How many patients are currently being followed up by remote monitoring in your centre?
   A) <50
   B) 50-200
   C) 201-500
   D) 501-1000
   E) >1000

10) Which remote monitoring systems are used in your centre?
   A) Abbott Merlin.net system
   B) Biotronik Home Monitoring system
   C) Boston Scientific Latitude Patient Management System
   D) Medtronic CareLink Network
   E) MicroPort Smartview system
   F) Medico Ermes

11) According to the type of device and patient'characteristics, to which patients is remote monitoring most frequently offered in your centre?
   A) All patients
   B) Patients with ICDs
   C) Patients with CRT devices
   D) Patients with PMs
   E) Patients with ILRs
   F) Patients with complex clinical issues
   G) Patients undergoing arrhythmia ablation procedures
   H) Patients with CIEDs at risk of malfunction
   I) Patients who live far from the hospital

12) What is the main purpose of remote monitoring in your centre?
   A) Scheduled remote device interrogation, in addition to automatic alerts for device/lead malfunction and for clinical events
   B) Scheduled remote device interrogation, in addition to automatic alerts for device/lead malfunction
   C) Complete replacement of in-office device checks

13) Which of the following alarms are considered critical in your centre?
   A) Lead malfunction
   B) ERI/EOL reached
   C) AHRE>6 h
   D) AHREs regardless of duration
   E) 1 VT/FV treated episode
   F) >1 VT/VF treated episode
   G) >1 NSVT episode
   H) Device related alarms
   I) Heart failure alarms

14) In your centre, has remote monitoring of PMs changed the frequency of in-office device follow-up visits?
A) No
B) Yes, we now perform in-office device follow-up examinations once a year
C) Yes, we now perform in-office device follow-up examinations less than once a year

15) In your centre, has remote monitoring of ICDs changed the frequency of in-office device follow-up visits?
A) No
B) Yes, we now perform in-office device follow-up examinations once a year
C) Yes, we now perform in-office device follow-up examinations less than once a year

16) In your centre, has remote monitoring of CRT devices changed the frequency of in-office device follow-up visits?
A) No
B) Yes, we now perform in-office device follow-up examinations once a year
C) Yes, we now perform in-office device follow-up examinations less than once a year

Organization of remote monitoring

17) Please indicate the professionals involved in the management of remote monitoring:
A) Physicians
B) Nurses
C) Cardiac physiologists (technicians)
D) Technical personnel from manufacturers
E) Others

18) Please indicate the frequency with which transmissions are reviewed in your centre:
A) <24 hours
B) Once a day (only on working days)
C) Once a week
D) Once every 2 weeks
E) Once a month
F) Other

19) Please indicate the professionals assigned to the primary review of remote data:
A) Physicians
B) Nurses
C) Cardiac physiologists (technicians)
D) Technical personnel from manufacturers
E) Others

20) When the primary review of transmissions is not performed by a physician, in which cases are the transmissions submitted to a physician?
A) In all cases
B) Only in the case of critical events
C) In no case

21) How is the patient informed of the results of remote transmissions?
A) Only in the case of clinically significant events

B) By letter
C) By phone
D) By sms (text message)
E) By email
F) Other

22) In your centre, do you usually share the clinical data collected by remote monitoring with other medical specialists?
A) No
B) With the family doctor
C) With the attending cardiologist
D) With other medical specialists

23) If so, how often are the reports sent?
A) When a critical event is detected
B) Once a year
C) Every 3-6 months

24) In your centre, is there an electronic health record to record remote clinical data and to make them available to all professionals involved in patient management?
A) Yes
B) No

<u>Impact of COVID-19 pandemic on the use of remote monitoring</u>

25) Was there an increase in the number of patients followed up by remote monitoring during COVID-19 pandemic?
A) Yes
B) No

26) If so, what was the increase?
A) <10%
B) 10-30%
C) 31-60%
D) 61-90%
E) >90%

27) If not, why was there no increase?
A) The need was not perceived
B) Organizational issues
C) Personnel shortage
D) Lack of a reimbursement system
E) Other reasons

28) Was a telehealth visits service as a replacement for in-person outpatient visits (in patients with or without CIED) activated during the COVID-19 lockdown period?
A) Yes
B) No

29) If so, how were the remote visits performed?
A) Phone calls
B) Video calls
C) Dedicated software

30) Will the telehealth visits service also be maintained in 2021?
A) Yes
B) No

31) Has a teleconsulting system for the family doctor or for physicians of other centres been activated in your center?
A) Yes
B) No

<u>Future perspectives</u>

32) What do you think is the main barrier to the implementation of remote monitoring for all CIED patients?
A) The lack of a reimbursement system
B) The excessive workload
C) Medical-legal issues
D) Issues related to the use of technology
E) Other reasons

33) In your opinion, how many patients will be followed up by remote monitoring in your centre in the next 5 years?
A) none
B) <10%
C) 10-30%
D) 31-60%
E) 61-90%
F) >90%

List of abbreviations
AHRE: atrial high rate episode
CIED: cardiac implantable electronic device
CRT-D cardiac resynchronization therapy combined with defibrillator
CRT-P cardiac resynchronization therapy combined with pacemaker
EOL: end-of-life
ERI: elective replacement indicator
ICD: implantable cardioverter-defibrillator
ILR: implantable loop recorder
NSVT: nonsustained ventricular tachycardia
PM: pacemaker
VF: ventricular fibrillation
VT: ventricular tachycardia.
